# Characterisation of macrophages infiltrating human mammary carcinomas.

**DOI:** 10.1038/bjc.1985.20

**Published:** 1985-01

**Authors:** R. J. Steele, M. Brown, O. Eremin


*Br. J. Cancer* (1985), **51**, 135–138

**Short Communication**

### Characterisation of macrophages infiltrating human mammary carcinomas

R.J.C. Steele, M. Brown & O. Eremin

*University Department of Clinical Surgery, Royal Infirmary, Lauriston Place, Edinburgh, EH3 9YW, UK.*

It is well established that most malignant tumours contain a variable number of macrophages (Evans, 1977; Russell *et al.*, 1981), but their presence in human breast cancer has been poorly documented. Although Wood & Gollahon (1977) demonstrated Fc(IgG) receptor-bearing cells in breast tumours, some investigators believe that malignant cells can express such receptors (Tonder & Thunold, 1973; Svennevig & Andersson, 1982). Morphologically, macrophages are not readily delineated in tissue sections of carcinomas, and in tumour cell suspensions precise identification is likewise difficult. This necessitates the use of cell markers, and in the present study, macrophages were identified by their ability to express receptors for Fc(IgG) and C3 and to possess HLA-DR antigens. In order to characterise these cells more precisely, a monoclonal antibody reactive with a myelomonocytic antigen (Kraft *et al.*, 1979) and a polyclonal antibody reactive with epithelial membrane antigen (EMA) (Heyderman *et al.*, 1979), were also employed.

Thirteen women with histologically confirmed primary invasive mammary carcinoma, clinically confined to the breast and axilla were studied. A portion of the carcinoma removed at operation was cleared of fat and fascia and washed in tissue culture medium (TCM). TCM consisted of RPMI-1640 (Gibco, Europe) with 10% heat-inactivated foetal calf serum, streptomycin ($100\,ug\,ml^{-1}$), penicillin G ($100{,}000\,i.u.\,ml^{-1}$), 0.7 g sodium bicarbonate $l^{-1}$ and 25 mM HEPES buffer. The tumour specimen was diced in TCM, and the pieces incubated in 15 mls collagenase (Sigma, Type I, $300\,U\,ml^{-1}$-dissolved in TCM without serum) at 37°C for 12 h with occasional agitation. The cell suspension was filtered through sterile gauze layers and washed six times in TCM to remove residual debris and tissue fragments. The cells were then resuspended in TCM at $2\times10^6\,ml^{-1}$, and cell numbers and viability were assessed using phase contrast microscopy (Eremin *et al.*, 1982).

Four surface markers were studied: the receptors for the Fc portion of IgG and for C3, HLA-DR antigen, identified by the mouse monoclonal antibody BT 2/9, and a macrophage-associated antigen defined by the mouse monoclonal antibody VEP-7. Fc(IgG) receptors were detected by means of the EA-rosetting reaction. Briefly, ox erythrocytes (E), were incubated with a subagglutinating but optimal dose of rabbit IgG anti-ox erythrocyte antiserum (A) (1:100 in PBS) for 45 min at room temperature (RT) (Eremin *et al.*, 1976). The sensitised EA indicator cells (2% suspension in TCM) were mixed with equal volumes of the tumour cell suspensions ($2\times10^6\,cells\,ml^{-1}$) and spun down. The pellet was allowed to stand at 4°C for 30 min and then resuspended by slow, continuous rotation for 1 min. C3 receptors were demonstrated using the EAC-rosetting reaction. Ox erythrocytes (E) were incubated with a subagglutinating but optimal dose of rabbit IgM anti-ox erythrocyte antibody (A) (1:40 in PBS) for 15 min at 4°C and then with an equal volume of C5 deficient mouse serum (1:10 in complement fixation test diluent) for 15 min at 37°C (Eremin *et al.*, 1976). The sensitised EAC indicators were then used in rosetting reactions as described above. Appropriate controls (heat-inactivated complement, EA-IgM) were set up and found to be negative. Fc(IgM) activity was absent in TCM (Eremin *et al.*, 1982). The HLA-DR antigen and the macrophage-associated antigen were detected by the direct antiglobulin rosetting reaction (DARR), as detailed previously (Coombs *et al.*, 1977). In this assay, the appropriate antibody was coupled, using 0.02% chromic chloride to trypsin-treated sheep erythrocytes. The latter were slowly rotated at RT for 60 min, washed in PBS, made up to a 1% suspension in PBS and stored at 4°C. The efficacy of the coupling was checked by a

Correspondence: R.J.C. Steele.
Received 11 June 1984; and in revised form 9 October 1984.

© The Macmillan Press Ltd., 1984

reverse passive haemagglutination test using rabbit anti-mouse immunoglobulin antibody (Dako). The cells to be tested ($2 \times 10^6 ml^{-1}$ in PBS) were mixed with an equal volume of indicator cells, spun down and allowed to stand at 4°C for 30 min.

Cytocentrifuge preparations of the rosetted cell suspensions were made and fixed in absolute ethanol for exactly 5 min. To detect cells expressing epithelial membrane antigen (EMA) in these preparations, the peroxidase-antiperoxidase method was used (Burns, 1975). A rabbit anti-human EMA antibody, diluted 1:400 in 5 ml of Tris-buffered saline containing 2% normal swine serum, was employed as the primary antibody, followed by a swine anti-rabbit immunoglobulin (Dako), and then by rabbit immunoglobulin against horseradish peroxidase conjugated with peroxidase (Dako). The distinctive reddish-brown colour, indicating the presence of the peroxidase, was produced by 3-amino 9-ethylcarbazole, diluted 1:10 with acetate buffer and one drop of 30 vol $H_2O_2$. The slides were incubated in this reagent for 5 min at RT and counterstained using haematoxylin. On each of the immunoperoxidase-stained cytocentrifuge preparations, 100 non-lymphocytic cells (assessed morphologically) were counted and the number rosetting was noted; the numbers of EMA-positive cells in the rosetted and non-rosetted populations were also recorded.

In order to assess the effect of collagenase in the preparative technique used to isolate the tumour macrophages, $1 \times 10^7$ mononuclear cells isolated on Ficoll-Hypaque from the blood of 10 healthy volunteers were incubated with 15 ml of collagenase for 12 h, washed six times in TCM and resuspended. Cell numbers and viability were assessed and the monocyte surface receptor-markers were then determined, as outlined above, on treated and non-treated cells.

The percentages of non-lymphocytic cells (15 to 40 $\mu$m) displaying Fc (IgG) receptors, HLA-DR antigens and VEP-7-defined antigens were substantial and comparable (Table I). C3 receptor expression, however, was less prevalent – see below and Eremin *et al.* (1982). Rosette-forming cells (>5 red blood cells attached to mononuclear cells) rarely stained for EMA (0–4%). A variable but significant number of non-rosetting cells, on the other hand, stained for EMA (Table II). A substantial number of cells lacked macrophage characteristics and EMA, probably reflecting differential expression of the latter and the presence of stromal (non epithelial) cells. With all four

**Table I** Percentage of macrophages in human breast tumour cell preparations

| *Macrophage receptor-marker* | *% Cells in breast tumour preparations expressing receptor-marker*[a,b] |
|---|---|
| Fc(IgG) receptor | 36.2±15.1 (15–59) |
| C3 receptor | 20.7±11.2 (4–40) |
| HLA-DR antigen | 40.8±11.8 (22–60) |
| VEP-7 antigen[c] | 39.7±13.9 (19–64) |

[a]Breast tumour cell suspensions obtained by digestion of tumour pieces with collagenase (see Text).
[b]Values expressed as mean±standard deviation (range)
[c]VEP-7 antigen: myelomonocyte-associated antigen detected by mouse monoclonal antibody (Kraft *et al.*, 1979).

**Table II** Percentage of cells expressing surface receptor-markers and EMA

| *Surface receptor-marker* | *% Cells in breast tumour preparations expressing receptor-marker and EMA*[a] | |
|---|---|---|
| | *EMA+* | *EMA−* |
| Fc(IgG)+ | 0.3± 0.6 (0–4) | 35.9±14.9 (15–59) |
| Fc(IgG)− | 20.6±13.5 (3–47) | 42.2±13.6 (24–78) |
| $C3^+$ | 0 | 20.7±11.2 (4–40)[b] |
| $C3^-$ | 20.4±11.1 (3–38) | 59.7± 9.9 (41–80) |
| HLA-DR+ | 0.2± 0.4 (0–4) | 40.7±11.6 (22–60) |
| HLA-DR− | 17.5±12.9 (0–40) | 41.6±13.7 (18–74) |
| VEP-7+ | 0.5± 0.7 (0–2) | 39.2±13.9 (19–64) |
| VEP-7− | 18.6±12.1 (3–43) | 41.2±13.6 (18–73) |

[a]Values expressed as mean±standard deviation (range).
[b]Low level of C3 receptor-bearing subset due to removal or modification of C3 receptor by collagenase (Eremin *et al.*, 1982).

assays, the asociation between EMA and the absence of rosette-forming ability was highly significant by Cox's precedure for combining several regressions which have a binary response (Cox, 1970). It should be noted that in the few cases where rosetted cells were positive for EMA, the staining exhibited a characteristic pattern, suggesting previous phagocytosis of EMA positive particles, rather than the diffuse cytoplasmic distribution seen in non-rosetted cells.

Phagocytosis of red cells was noted on the cytocentrifuge preparations in 5–15% of the Fc(IgG) receptor-positive cells. Prior incubation of these cells at 37°C for 2 h however, led to a marked increase in the incidence of phagocytosis, and ingested erythrocytes were seen in 20-60% of rosetted cells.

Pre-incubation of blood monocytes with collagenase did not markedly alter the expression of Fc(IgG) receptors, HLA-DR antigens or VEP-7-defined antigen expression. EAC rosette formation, however, was inhibited, indicating that C3 receptors were stripped off or altered by the collagenase preparation. The latter contained small amounts of trypsin which is known to modify the surface receptor for C3 (Henson, 1969), although prolonged incubation at 37°C can lead to re-expression of the receptor (Eremin *et al.*, 1982). Only small, nonspecific cell losses occurred during incubation with collagenase (Eremin *et al.*, 1982).

This study has shown that human breast cancers contain a substantial population of non-lymphocytic, medium to large (15 to 40 $\mu$m) cells bearing Fc(IgG) receptors, C3 receptors, HLA-DR and macrophage-associated antigens. Macrophages are known to bear Fc(IgG) receptors (Berken & Benacerraf, 1966; Nelson, 1981) and to express HLA-DR antigens (Hirchberg *et al.*, 1976). Morphologically, the rosetting cells in the present study resembled macrophages and a variable percentage contained phagocytosed indicator red blood cells. In addition, the numbers of cells displaying Fc(IgG) receptors and HLA-DR antigen were comparable to the numbers bearing the VEP-7-defined antigen, suggesting that these cells were macrophages. The C3 receptor-bearing cells, however, were fewer in number than the VEP-7-positive cells, a phenomenon probably due to removal or alteration of the glycoprotein receptor for C3 by trypsin in the collagenase preparation. Thus, in view of the documented presence of C3 receptors on macrophages (Huber *et al.*, 1968; Nelson, 1981), the morphological appearance of the rosetting cells and the observed phagocytosis of the EAC cells, the C3 receptor-positive cells isolated from the tumours were probably also macrophages.

These observations alone, however, do not establish unequivocally that the cells bearing Fc or C3 receptors and HLA-DR antigens are macrophages. Although the Fc(IgG) receptor has been widely used as a macrophage marker in tumour cell suspensions (Russell *et al.*, 1981), some workers have suggested that malignant cells themselves may express Fc(IgG) receptors (Tonder & Thunold, 1973; Svennevig & Andersson, 1982). In addition, it has been shown that human monocytes can shed these receptors in culture (Kay & Douglas, 1981), raising the possibility of non-specific receptor uptake by tumour cells. HLA-DR antigens are certainly found on macrophages and other antigen-presenting cells, but some lymphocyte subsets also display them (Barclay & Mason, 1983). A previous study, however, has documented low numbers only of activated T cells and B lymphoblasts within breast carcinomas (Eremin *et al.*, 1982), suggesting that these cells are an unlikely source of error. Recently some workers have demonstrated HLA-DR antigens in tissue sections of human tumours (Natali *et al.*, 1981; Gatter *et al.*, 1982), but in these reports, no attempt was made to differentiate the tumour-infiltrating macrophages from the neoplastic cells.

The present study, on the other hand, has established that the malignant cells in the breast tumour cell suspensions studied did not express the receptors-markers employed to characterise macrophages. Rabbit anti human EMA (Heyderman *et al.*, 1979), was used to identify epithelial cells in cytocentrifuge preparations of breast tumour cell suspensions in which macrophages were delineated by rosette formation. EMA is expressed by nearly all breast cancers (Dearnaley *et al.*, 1981), but it is not present on all cells within a single tumour (Heyderman *et al.*, 1979) and it is not exclusive to neoplastic cells. EMA is only expressed by epithelial cells, however (Sloane & Ormerod, 1981), and in suspensions from breast cancers, EMA positive cells can be assumed to be neoplastic.

In summary, this study has shown firstly that human breast carcinomas contain substantial, although variable, numbers of macrophages (19–64%). Secondly the study suggests that the majority of these tumour-infiltrating macrophages express Fc(IgG) and C3 receptors and HLA-DR antigens. Finally, using an antibody reactive with EMA, it has been demonstrated that breast carcinoma cells lack these macrophage receptor-markers.

We thank Prof. A.P.M. Forrest of the University Department of Clinical Surgery for allowing us to study patients under his care and Prof. Sir Alastair Currie for access to pathologic specimens. The monoclonal antibody BT 2/9 was a gift from Dr. H. Waldman (Cambridge) and the monoclonal antibody VEP-7 was a gift from Dr. D. Kraft (Vienna). The rabbit polyclonal antibody reactive with EMA was a gift from Dr. Ormerod (London).

The work was supported by a grant from the Cancer Research Campaign (UK).

### References

BARCLAY, A.N. & MASON, D.W. (1983). Graft rejection, and Ia antigens-paradox resolved? *Nature*, **303**, 382.

BERKEN, A. & BENACERRAF, B. (1966). Properties of antibodies cytophilic for macrophages. *J. Exp. Med.*, **123**, 119.

BURNS, J. (1975). Background staining and sensitivity of the unlabelled antibody-enzyme (PAP) method. Comparison with the peroxidase-labelled antibody sandwich method using formalin-fixed paraffin embedded material. *Histochemistry*, **43**, 291.

COOMBS, R.R., WILSON, A.B., EREMIN, O. & 5 others. (1977). Comparison of the direct antiglobulin rosetting reaction with the mixed antiglobulin rosetting reaction for the detection of immunoglobulin on lymphocytes. *J. Immunol Methods*, **18**, 45.

COX, D.R. (1970). *Analysis of Binary Data*. Methuen, London. p. 65.

DEARNALEY, D.P., SLOANE, J.P., ORMEROD, M.G. & 7 others. (1981). Increased detection of mammary carcinoma cells in marrow smears using antisera to epithelial membrane antigen. *Br. J. Cancer*, **44**, 85.

EREMIN, O., PLUMB, D. & COOMBS, R.R. (1976). T and B lymphocyte populations in human normal lymph node, regional tumour lymph node and inflammatory lymph node. *Int. Arch Allergy Appl Immunol.*, **52**, 277.

EREMIN, O., COOMBS, R.R.A., PROSPERO, T.D. & PLUMB, D. (1982). T-lymphocyte and B-lymphocyte subpopulations infiltrating human mammary carcinomas. *J. Natl Cancer Inst.*, **69**, 1.

EVANS, R. (1977). Macrophages in solid tumours. In: *The Macrophage and Cancer*. (Eds: James *et al.*) Econoprint, Edinburgh p. 321.

GATTER, K.C., ABDULAZIZ, Z., BEVERLEY, P. & 10 others. (1982). Use of monoclonal antibodies for the histopathological diagnosis of human malignancy. *J. Clin. Pathol.*, **35**, 1253.

HENSON, P.M. (1969). The adherence of leucocytes and platelets induced by fixed IgG antibody or complement. *Immunology*, **16**, 107.

HEYDERMAN, E., STEELE, K. & ORMEROD, G. (1979). A new antigen on the epithelial membrane: its immunoperoxidase localisation in normal and neoplastic tissue. *J. Clin. Pathol.*, **32**, 35.

HIRCHBERG, H., KAAKINEN, A. & THORSBY, E. (1976). Presence of HLA-D determinants on human macrophages. *Nature*, **263**, 63.

HUBER, H., POLLEY, M.J., LINSCOTT, W.D., FUDENBERG, H.H. & MULLER-EBERHARD, H.J. (1968). Human monocytes: distinct receptor sites for the 3rd component of complement and for IgG. *Science*, **162**, 1281.

KAY, N.E. & DOUGLAS, S.D. (1981). Detection of shedding of human blood monocyte Fc receptor during in vitro culture. *Int. Arch Allergy Appl. Immunol.*, **66**, 131.

KRAFT, D., RUMPOLD, K., STEINER, R. & 4 others. (1979). Evidence against a myeloid nature of human large granular lymphocytes (LGL). In: *Mechanisms of Lymphocyte Activation*. (Eds Resch & Kirchner) Elsevier/North Holland Biomedical Press. Amsterdam, p. 279.

NATALI, P.G., DE MARTINO, C., QUARANTA, V., BIGOTTI, A., PELLEGRINO, M.A. & FERRONE, S. (1981). Changes in Ia-like antigen expression on malignant human cells. *Immunogenetics*, **12**, 409.

NELSON, D.S. (1981). Macrophages: progress and problems. *Clin. Exp. Immunol.*, **45**, 225.

RUSSELL, S.W., GILLESPIE, G.Y. & PACE, J.L. (1981). Evidence for mononuclear phagocytes in solid neoplasms and appraisal of their non-specific cytotoxic capabilities. *Contemp. Topics in Immunobiol*, **10**, 143.

SLOANE, J.P. & ORMEROD, M.G. (1981). Distribution of epithelial membrane antigen in normal and neoplastic tissues. *Cancer*, **47**, 1786.

SVENNEVIG, J.-L. & ANDERSSON, T.R. (1982). Cell bearing Fc receptors in human malignant solid tumours. *Br. J. Cancer*, **45**, 201.

TONDER, O. & THUNOLD, S., (1973). Receptors for immunoglobulin Fc in human malignant tumours. *Scand. J. Immunol.*, **2**, 207.

WOOD, G.W. & GOLLAHON, K.A. (1977). Detection and quantitation of macrophage infiltration into primary human tumours with the use of cell-surface markers. *J. Natl Cancer Inst.*, **59**, 1081.


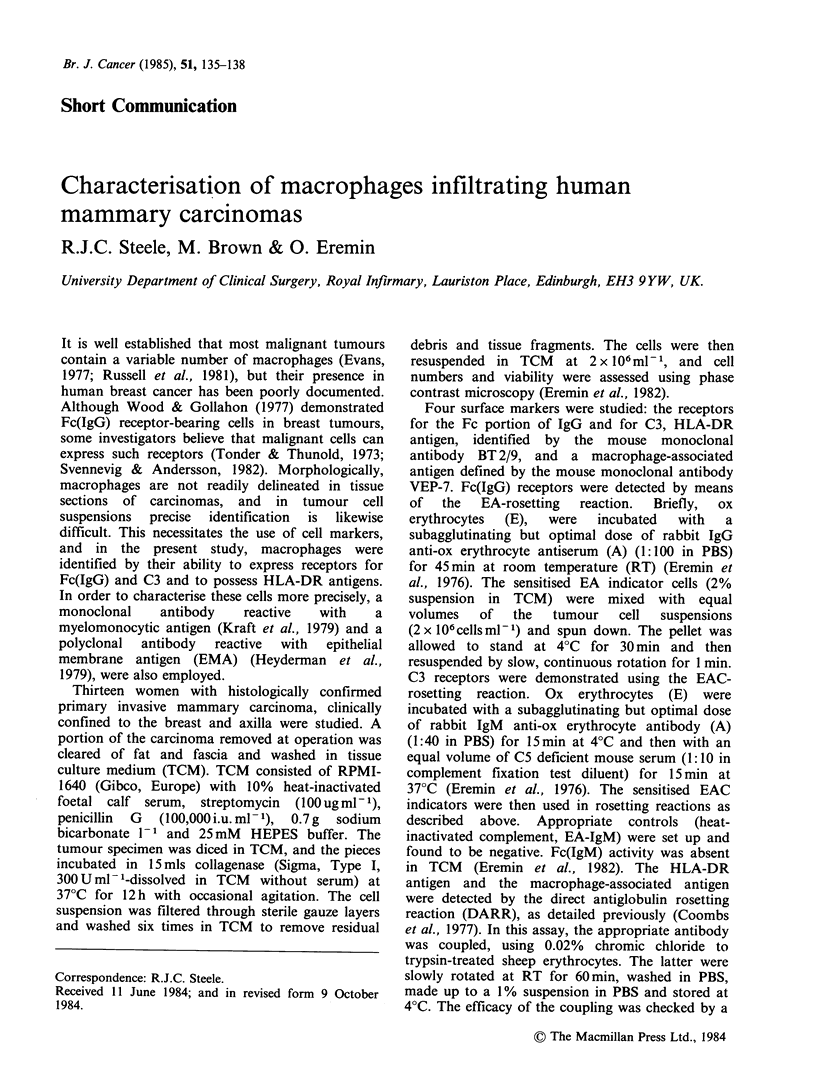

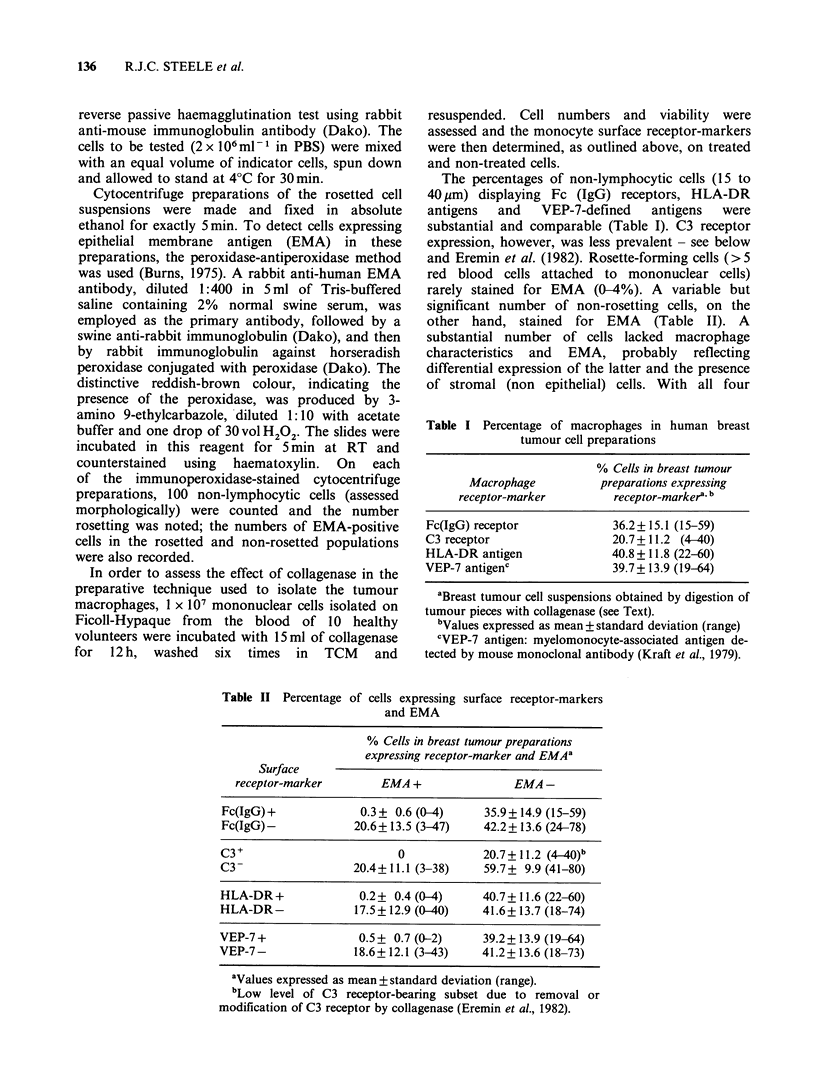

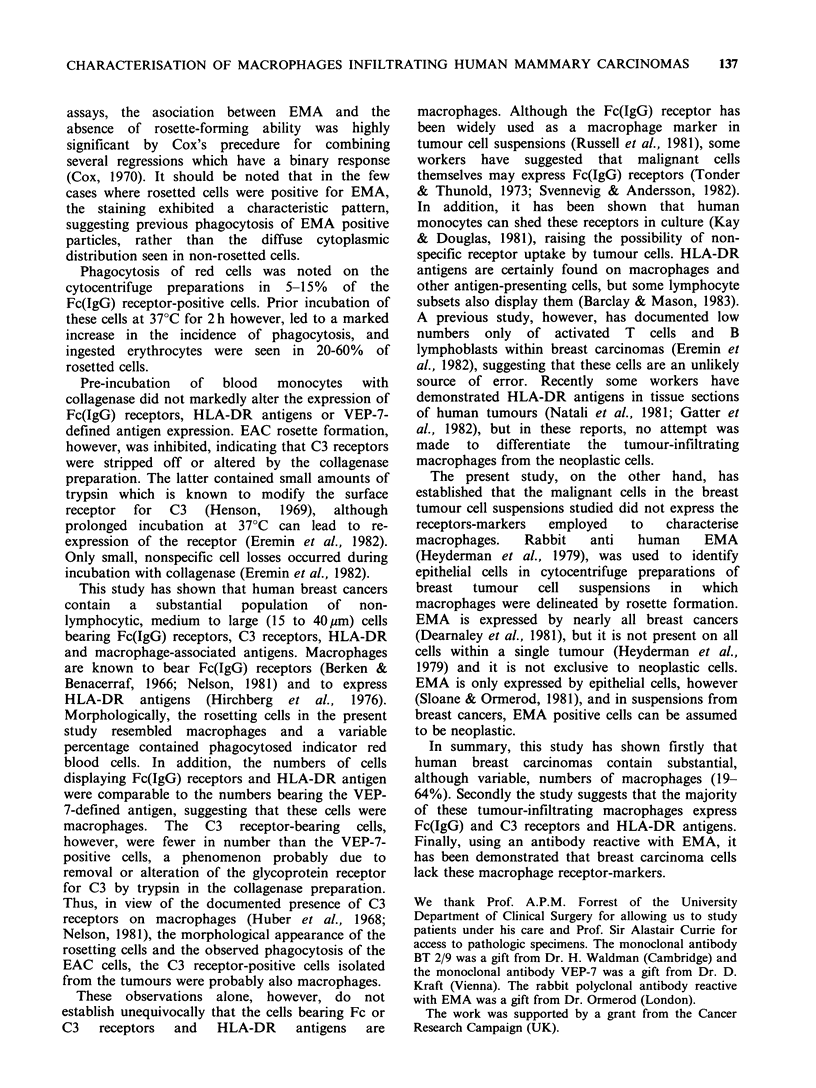

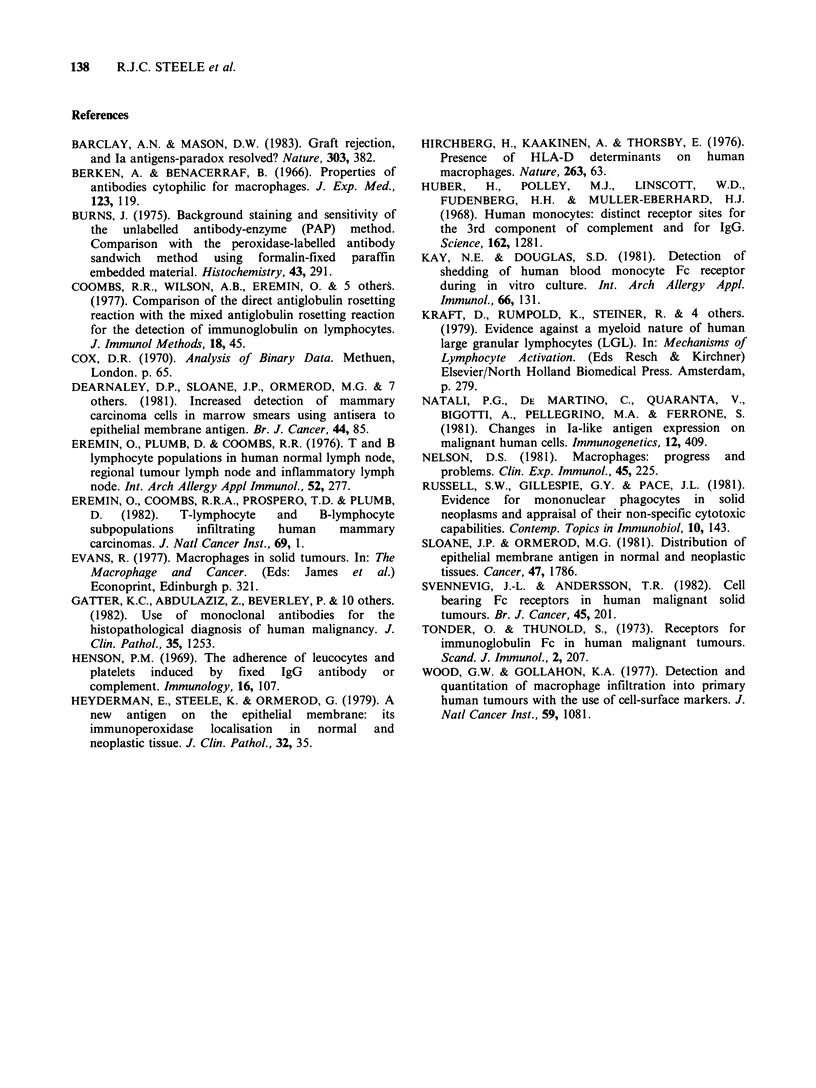


## References

[OCR_00349] Barclay A. N., Mason D. W. (1983). Graft rejection and Ia antigens--paradox resolved?. Nature.

[OCR_00353] Berken A., Benacerraf B. (1966). Properties of antibodies cytophilic for macrophages.. J Exp Med.

[OCR_00358] Burns J. (1975). Background staining and sensitivity of the unlabelled antibody-enzyme (PAP) method. Comparison with the peroxidase labelled antibody sandwich method using formalin fixed paraffin embedded material.. Histochemistry.

[OCR_00388] Eremin O., Coombs R. R., Prospero T. D., Plumb D. (1982). T-lymphocyte and B-lymphocyte subpopulations infiltrating human mammary carcinomas.. J Natl Cancer Inst.

[OCR_00382] Eremin O., Plumb D., Coombs R. R. (1976). T and B lymphocyte populations in human normal lymph node, regional tumour lymph node and inflammatory lymph node.. Int Arch Allergy Appl Immunol.

[OCR_00399] Gatter K. C., Abdulaziz Z., Beverley P., Corvalan J. R., Ford C., Lane E. B., Mota M., Nash J. R., Pulford K., Stein H. (1982). Use of monoclonal antibodies for the histopathological diagnosis of human malignancy.. J Clin Pathol.

[OCR_00405] Henson P. M. (1969). The adherence of leucocytes and platelets induced by fixed IgG antibody or complement.. Immunology.

[OCR_00410] Heyderman E., Steele K., Ormerod M. G. (1979). A new antigen on the epithelial membrane: its immunoperoxidase localisation in normal and neoplastic tissue.. J Clin Pathol.

[OCR_00416] Hirschberg H., Kaakinen A., Thorsby E. (1976). Presence of HLA-D determinants on human macrophages.. Nature.

[OCR_00421] Huber H., Polley M. J., Linscott W. D., Fudenberg H. H., Müller-Eberhard H. J. (1968). Human monocytes: distinct receptor sites for the third component of complement and for immunoglobulin G.. Science.

[OCR_00428] Kay N. E., Douglas S. D. (1981). Detection of shedding of human blood monocyte Fc receptor during in vitro culture.. Int Arch Allergy Appl Immunol.

[OCR_00442] Natali P. G., De Martino C., Quaranta V., Bigotti A., Pellegrino M. A., Ferrone S. (1981). Changes in Ia-like antigen expression on malignant human cells.. Immunogenetics.

[OCR_00448] Nelson D. S. (1981). Macrophages: progress and problems.. Clin Exp Immunol.

[OCR_00452] Russell S. W., Gillespie G. Y., Pace J. L. (1980). Evidence for mononuclear phagocytes in solid neoplasms and appraisal of their nonspecific cytotoxic capabilities.. Contemp Top Immunobiol.

[OCR_00458] Sloane J. P., Ormerod M. G. (1981). Distribution of epithelial membrane antigen in normal and neoplastic tissues and it value in diagnostic tumor pathology.. Cancer.

[OCR_00463] Svennevig J. L., Andersson T. R. (1982). Cells bearing Fc receptors in human malignant solid tumours.. Br J Cancer.

[OCR_00468] Tonder O., Thunold S. (1973). Receptors for immunoglobulin Fc in human malignant tissues.. Scand J Immunol.

[OCR_00473] Wood G. W., Gollahon K. A. (1977). Detection and quantitation of macrophage infiltration into primary human tumors with the use of cell-surface markers.. J Natl Cancer Inst.

